# Megalencephalic Leukoencephalopathy: Insights Into Pathophysiology and Perspectives for Therapy

**DOI:** 10.3389/fncel.2020.627887

**Published:** 2021-01-22

**Authors:** Assumpció Bosch, Raúl Estévez

**Affiliations:** ^1^Department of Biochemistry and Molecular Biology, Institute of Neurosciences, Univ. Autònoma de Barcelona, Barcelona, Spain; ^2^Unitat Mixta UAB-VHIR, Vall d’Hebron Institut de Recerca (VHIR), Barcelona, Spain; ^3^Centro de Investigación Biomédica en Red sobre Enfermedades Neurodegenerativas (CIBERNED), Instituto de Salud Carlos III, Madrid, Spain; ^4^Departament de Ciències Fisiològiques, IDIBELL—Institute of Neurosciences, Universitat de Barcelona, Barcelona, Spain; ^5^Centro de Investigación Biomédica en Red sobre Enfermedades Raras (CIBERER), Instituto de Salud Carlos III, Madrid, Spain

**Keywords:** myelin abnormalities, ion channel, water homeostasis, chloride, cell-cell adhesion

## Abstract

Megalencephalic leukoencephalopathy with subcortical cysts (MLC) is a rare genetic disorder belonging to the group of vacuolating leukodystrophies. It is characterized by megalencephaly, loss of motor functions, epilepsy, and mild mental decline. In brain biopsies of MLC patients, vacuoles were observed in myelin and in astrocytes surrounding blood vessels. It is mainly caused by recessive mutations in *MLC1* and *HEPACAM* (also called *GLIALCAM*) genes. These disease variants are called MLC1 and MLC2A with both types of patients sharing the same clinical phenotype. Besides, dominant mutations in *HEPACAM* were also identified in a subtype of MLC patients (MLC2B) with a remitting phenotype. MLC1 and GlialCAM proteins form a complex mainly expressed in brain astrocytes at the gliovascular interface and in Bergmann glia at the cerebellum. Both proteins regulate several ion channels and transporters involved in the control of ion and water fluxes in glial cells, either directly influencing their location and function, or indirectly regulating associated signal transduction pathways. However, the MLC1/GLIALCAM complex function and the related pathological mechanisms leading to MLC are still unknown. It has been hypothesized that, in MLC, the role of glial cells in brain ion homeostasis is altered in both physiological and inflammatory conditions. There is no therapy for MLC patients, only supportive treatment. As MLC2B patients show an MLC reversible phenotype, we speculated that the phenotype of MLC1 and MLC2A patients could also be mitigated by the re-introduction of the correct gene even at later stages. To prove this hypothesis, we injected in the cerebellar subarachnoid space of *Mlc1* knockout mice an adeno-associated virus (AAV) coding for human MLC1 under the control of the glial-fibrillary acidic protein promoter. MLC1 expression in the cerebellum extremely reduced myelin vacuolation at all ages in a dose-dependent manner. This study could be considered as the first preclinical approach for MLC. We also suggest other potential therapeutic strategies in this review.

## Introduction

Leukodystrophies are human diseases affecting the central nervous system (CNS) myelin. Although individual leukodystrophies are rare, epidemiological data are indicating their high incidence. Within the leukodystrophies, Megalencephalic leukoencephalopathy with subcortical cysts (MLC; MIM 604004) is a vacuolating white matter disorder of infantile-onset (van der Knaap et al., 2012). Macrocephaly is observed in all affected individuals and develops during the first year of life. Most patients show delayed mental or motor development and seizures (Hamilton et al., 2018). Magnetic resonance imaging (MRI) is crucial to diagnose the disease in childhood, showing diffuse signal abnormality, swelling of the cerebral white matter, and the presence of subcortical cysts, which are mostly present in the anterior temporal areas and often in the frontoparietal region (van der Knaap et al., 1995).

From this initial presentation, which is common to all MLC patients, two different phenotypes have been described: a classical phenotype, which is found in the majority of patients, and an improving phenotype, which shows a bettering clinical course (van der Knaap et al., 2010). In the MLC classical phenotype, there is a gradual onset of ataxia, spasticity, and sometimes extrapyramidal findings; and late-onset of mild mental deterioration. In the improving phenotype, motor function ameliorates or normalizes; some patients have stable intellectual disability, with or without autism (Hamilton et al., 2018).

The classical MLC can be caused by two biallelic mutations in the *MLC1* or *HEPACAM* genes, as the disease is inherited in a recessive manner (Leegwater et al., 2001; López-Hernández et al., 2011a). Classical MLC patients with mutations in *MLC1* are diagnosed as MLC1 type, whereas those with mutations in *HEPACAM* are named MLC2A. The improving phenotype, on the other hand, is caused by heterozygous mutations in *HEPACAM* (López-Hernández et al., 2011a). These patients are diagnosed as MLC2B type, and the disease is inherited dominantly. Mutations in *MLC1* and *HEPACAM* are found in 76% and 22% of the patients, respectively. Pathogenic variants in *MLC1* or *HEPACAM* have not been identified in the remaining 2% of individuals showing clinical features of MLC, suggesting the existence of unknown additional genes of the disease (van der Knaap et al., 1993).

*MLC1* encodes for an integral oligomeric membrane protein of unknown function expressed only in astrocytes, being one of the most specific proteins for this cellular type (Teijido et al., 2004, 2007; Boor et al., 2005; Ambrosini et al., 2008). Mutations found in MLC1 patients are distributed all over the protein and they cause protein instability, which leads to its degradation at the endoplasmic reticulum (ER) or in lysosomes (Duarri et al., 2008; Lanciotti et al., 2010; Petrini et al., 2013). *HEPACAM* encodes for a single transmembrane type I protein (Moh et al., 2005), which works as an adhesion molecule called HepaCAM or GlialCAM, the latter reflecting its higher expression in glial cells (Barrallo-Gimeno et al., 2015). GlialCAM can form homophilic interactions with other GlialCAM molecules within the same cell (*cis* interactions) and between different cells (*trans* interactions; Elorza-Vidal et al., 2020). Most missense mutations found in MLC2A or MLC2B patients are located in the extracellular domain of the protein, and affect protein localization at cell-cell junctions (López-Hernández et al., 2011a; Arnedo et al., 2014a,b; Elorza-Vidal et al., 2020). A recessive mutation in the signaling peptide of GlialCAM (p.L23H) abolishes protein expression in transfected cells *in vitro*, suggesting that the lack of GlialCAM expression can also cause the disease (Arnedo et al., 2014b). Based on recent biochemical and structural data, it seems that missense dominant MLC2B mutations specifically affect GlialCAM *cis* and *trans* interaction surfaces (Elorza-Vidal et al., 2020).

GlialCAM exerts two roles on MLC1: on one hand, it acts as an ER chaperone (Capdevila-Nortes et al., 2013) and, on the other hand, it confines MLC1 at astrocyte-astrocyte junctions (López-Hernández et al., 2011b). Thus, most MLC2A and MLC2B *HEPACAM* mutations affect GlialCAM targeting and, consequently, also MLC1 location to cell-cell junctions. As GlialCAM improves MLC1 folding, co-expression of GlialCAM with mutated MLC1 increases levels of MLC1 protein and its membrane expression (Capdevila-Nortes et al., 2013).

## Effect of MLC Mutations into Glialcam and MLC1 Cell Biology

Different cellular models have been used to express wild-type MLC1 and GlialCAM or containing MLC mutations, such as *Xenopus oocytes* (Teijido et al., 2004), *Spodoptera frugiperda* (Sf9) cells (Ridder et al., 2011) and other cell lines such as fibroblast HeLa (López-Hernández et al., 2011b), COS-1 (Hwang et al., 2019), HEK293 or glioblastoma U373 (Ambrosini et al., 2008) and human U251 astrocytoma (Lanciotti et al., 2020). Other studies have been performed in primary astrocyte cultures from mice, rats, and humans (Duarri et al., 2011; Ridder et al., 2011; Brignone et al., 2014). Finally, studies have also been performed in lymphoblast cell lines and human monocytes or monocyte-derived macrophages from control and MLC patients (Duarri et al., 2008; Petrini et al., 2013). Studies with human astrocytes derived from iPS cells would also be useful to gather more information on the disease.

**Figure 1:**
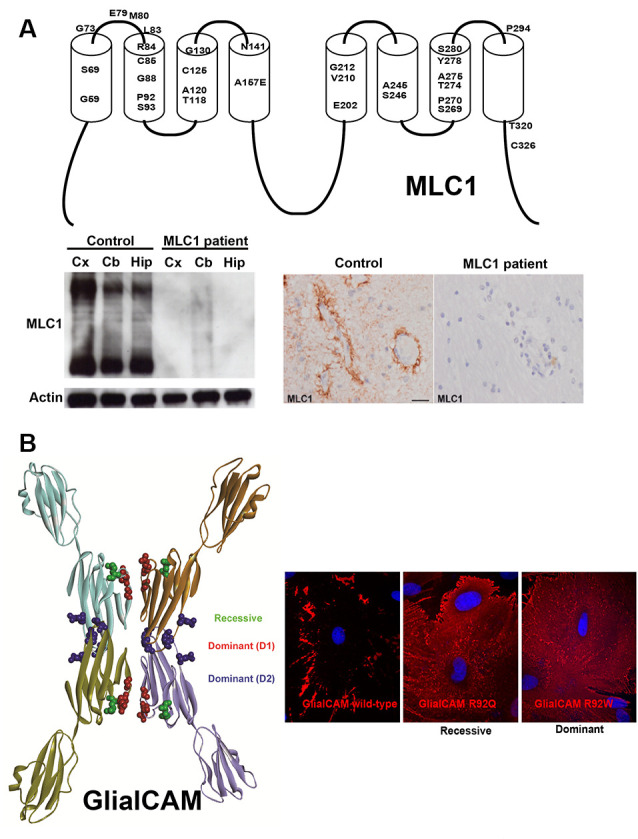
Effect of *MLC1* **(A)** and *GLIALCAM* **(B)** mutations on the biochemistry and cell biology of their coding proteins. **(A)** Top panel: 2D-scheme of the MLC1 protein showing the residues that are mutated in Megalencephalic leukoencephalopathy with subcortical cysts (MLC) patients. Below panel: mutation S69L found in a Spanish MLC1 patient abolished MLC1 protein expression. Reprinted by permission from Oxford University Press: Human Molecular Genetics, *Molecular mechanisms of MLC1 and GLIALCAM mutations in MLC*, López-Hernández et al. (2011b). **(B)** 3D-Model of GlialCAM homo-oligomers forming cis and trans interactions. Reprinted by permission from Oxford University Press: Human Molecular Genetics, *Structural basis for the dominant or recessive character of GLIALCAM mutations found in leukodystrophies*, Elorza-Vidal et al. (2020). Expression of GlialCAM containing a recessive (R92Q) or a dominant (R92W) mutation in primary astrocytes causes mistargeting.

From a pathological point of view, these studies in cell lines have defined the biochemical mechanism of how different MLC mutations affect different parameters such as protein stability, protein levels at the plasma membrane, and protein localization. Thus, it is clear that most *MLC1* missense mutations cause protein degradation. This has been verified in *Xenopus oocytes* (Duarri et al., 2008), different cell lines (Duarri et al., 2008; Brignone et al., 2014), primary astrocyte cultures (Duarri et al., 2008), monocytes from patients (Duarri et al., 2008; Petrini et al., 2013), and even in a brain biopsy of an MLC1 patient (López-Hernández et al., 2011b; Figure 1A). Therefore, cells or animals deficient in MLC1 constitute a model to understand the pathophysiology of MLC. Animal models containing a missense mutation in MLC1 could be used to screen for chemical chaperones that could increase MLC1 protein stability and consequently, its levels at the plasma membrane.

On the other hand, most mutations in *HEPACAM* cause protein mislocalization, as shown in cell lines and cultured astrocytes (Figure 1B). Biochemical studies also suggest that dominant MLC2B mutations affect the trafficking of the wild-type protein, possibly by interfering with the formation of GlialCAM complexes in the same cell (*cis*-interactions) or between different cells (*trans*-interactions; Elorza-Vidal et al., 2020). These *in vitro* studies were validated *in vivo* with the generation of a *Glialcam* knockin (KI) mouse containing a dominant mutation, which showed that GlialCAM protein also displays a trafficking defect in heterozygous mice (Hoegg-Beiler et al., 2014). With this model, it has been speculated that dominant MLC2B mutations may lead to a minor reduction of GlialCAM/MLC1 function, which might be pathogenic in humans only at the early stages of life. In agreement with the reversibility of the phenotype observed in MLC2B patients in adult stages, a decreased GlialCAM/MLC1 function may not be so pathogenic in adults as it is in infants (Hamilton et al., 2018).

Very few *HEPACAM* mutations seem to induce a gain of function, a phenotype that can only be observed in astrocytes from *Mlc1* knockout mice in depolarizing conditions (Arnedo et al., 2014b). Further work is needed to understand the molecular mechanism of the pathogenesis of these specific mutations.

## Insights into Glialcam/MLC1 Function and Pathophysiology of MLC

It has nearly been 20 years since the discovery of the first gene implicated with the disease, *MLC1* (Leegwater et al., 2001). However, we still do not have a clear picture of the function this protein exerts, and hence, the pathophysiology of the disease remains obscure (Estévez et al., 2018).

First insights into the putative role of MLC1 came from protein sequence analysis (Leegwater et al., 2001). Thus, it was suggested that MLC1 could work as an ion channel or a transporter. However, no ion or transport activity directly mediated by MLC1 has been demonstrated so far, even when co-expressed with GlialCAM (Teijido et al., 2004). Based on the clinical phenotype of the patients, it was then speculated that water/ion homeostasis should be negatively affected by the lack of MLC1 (van der Knaap et al., 2012).

To get more insights into the role of GlialCAM/MLC1, several research groups aimed to identify first the MLC1 and later GlialCAM interacting proteins. Different approaches were addressed such as yeast-to-hybrid, using MLC1 N-terminal domain (Brignone et al., 2011) or the full-length MLC1 (unpublished results from our group), immunopurification of MLC1 or GlialCAM associated complexes (López-Hernández et al., 2011a; Jeworutzki et al., 2012; Sugio et al., 2017), co-fractionation (sWGA and DEAE chromatography, ouabain chromatography; Ambrosini et al., 2008; Brignone et al., 2011, 2014), co-localization (by confocal or electronic microscopy immunogold) and by testing possible candidates (Lanciotti et al., 2012). All these studies resulted in a list of proteins that might be involved in MLC (Table 1).

**Table 1:** Proteins that have been related to the Megalencephalic leukoencephalopathy with subcortical cysts (MLC) proteins.

Protein related with MLC	Genetical, cell biology and biochemical evidence	Overexpression	Reduction of expression	Some comments
*GlialCAM*	1. Mutations in *GLIALCAM* cause MLC (López-Hernández et al., 2011a). 2. *GlialCAM* interact with MLC1 *in vitro*: co-IP, FRET, split-TEV (López-Hernández et al., 2011b). 3. Co-localization and co-IP *in vivo* (López-Hernández et al., 2011a). 4. GlialCAM change MLC1 localization (López-Hernández et al., 2011b).	Overexpression of GlialCAM stabilize MLC1 wild-type and mutants (Capdevila-Nortes et al., 2013).	1. KO of Glialcam affect MLC1 expression and localization (Hoegg-Beiler et al., 2014; Bugiani et al., 2017). 2. KO of *Mlc1* affect GlialCAM localization (Hoegg-Beiler et al., 2014; Bugiani et al., 2017).	There is no doubt that GlialCAM is a obligate subunit of MLC1 *in vivo*. However, MLC1 overexpressed *in vitro* arrive at the PM independently of GlialCAM.
*ClC-2*	1. ClC-2 interact with GlialCAM and MLC1 *in vitro*: coIP, split-TEV, stabilization at the PM (Jeworutzki et al., 2012; Gaitán-Peñas et al., 2017). 2. Co-localization and co-IP *in vivo* (Jeworutzki et al., 2012). 3. ClC-2 localization change by GlialCAM (Jeworutzki et al., 2012).	Overexpression of ClC-2 and GlialCAM change functional properties of ClC-2 (Jeworutzki et al., 2012, 2014).	1. KO of Mlc1 and Glialcam change ClC-2 localization and functional properties (Hoegg-Beiler et al., 2014; Bugiani et al., 2017). 2. In KO of ClC-2 there is a slight increase of MLC1 (Hoegg-Beiler et al., 2014).	The association between GlialCAM/MLC1 and ClC-2 is not constitutive (Sirisi et al., 2017). ClC-2 KO and/or mutations of CLCN2 have a different phenotype than MLC (Depienne et al., 2013). ClC-2 dysfunction might contribute to MLC but it is not the whole story (Hoegg-Beiler et al., 2014).
*Na^+^/K^+^-ATPase*	1. β1 subunit interact with *MLC1* N terminus by Yeast two-hybrid and pull down *in vitro* (Brignone et al., 2011). However, β1 was not co-IP *in vivo* (Sugio et al., 2017). 2. α2 and α3 subunits were co-IP *in vivo* (Sugio et al., 2017). 3. Some colocalization in astrocytes (Brignone et al., 2011). 4. Ouabain chromatography purified MLC1 (Brignone et al., 2011). 5. Other proteins copurifying with Na^+^/K^+^-ATPase also are found in MLC1 IP (i.e., GLAST, GLT-1; Sugio et al., 2017).	Astrocytes from mice with MLC1 OE show reduced Na^+^/K^+^-ATPase activity, but increased ouabain binding (Sugio et al., 2017).	1. No change in activity were observed in Mlc1 KO mice (Sugio et al., 2017). 2. No change in localization were observed in MLC KO mice (Dubey et al., 2015; Bugiani et al., 2017).	*In vitro* studies expressing these proteins are needed to prove their interaction. More evidence *in vivo* that Na^+^/K^+^-ATPase activity is regulated by MLC1 is necessary.
*Other ATPases: PMCA1, 2, 3; SERCA1, 2*; V-ATPase	1. They have been identified by coIP from brain tissue or by pulldown from astrocytes expressing His-tagged MLC1 (Brignone et al., 2014; Sugio et al., 2017). 2. Some co-localization have been demonstrated by IF in astrocytes with V-ATPase (Brignone et al., 2014).	In the case of V-ATPase: OE of MLC1 influences endosomal pH (Brignone et al., 2014).	No studies have been performed in KO animals.	*In vitro* studies expressing these proteins are needed to prove their interaction. More evidence *in vivo* that ATPase activity is regulated by MLC1 is necessary.
*VRAC channel (LRRC8A)*	1. No coIP *in vivo* (Elorza-Vidal et al., 2018). 2. No colocalization in tissue and in primary astrocytes (Elorza-Vidal et al., 2018).	OE MLC1 but not GlialCAM increases VRAC activity and improves RVD (regulatory volume decrease; Ridder et al., 2011; Lanciotti et al., 2012; Capdevila-Nortes et al., 2013).	1. *Mlc1* KO or *Mlc1/Glialcam* depletion reduces VRAC activity and RVD in astrocytes (Capdevila-Nortes et al., 2013; Dubey et al., 2015; Elorza-Vidal et al., 2018). 2. Lymphoblast MLC cell lines show reduced VRAC activity (although MLC1 mRNA expression is nearly negligible in WT cells; Ridder et al., 2011).	It is not clear how VRAC activity is regulated by MLC1. It may be an indirect or a compensatory mechanism. More experimental evidence is needed. For instance, VRAC current should be measured in brain slices from MLC KO models.
		
*Cx43*	1. coIP *in vivo* with GlialCAM (our unpublished results) and *in vitro* in U373G cells (Boor et al., 2007). 2. Partial co-localization with MLC1 and GlialCAM in primary astrocytes (Duarri et al., 2011).	1. OE MLC1 in U251 cell line reduces ERK1/2 activity which phosphorylates Cx43 increasing Cx43 in gap junctions (Lanciotti et al., 2020). 2. OE GlialCAM in U373G cell line increase Cx43 stability (Boor et al., 2007).	1. No experiments have been performed in KO animals. 2. Cx43 is misslocalized in *Glialcam* KO astrocytes (our unpublished results).	Experiments *in vivo* in KO MLC models are needed. It seems that MLC1 may influence different proteins indirectly by influencing signaling through ERK1/2 (VRAC, Cx43; Lanciotti et al., 2016).
*TRPV4*	1. It has been identified by pulldown from astrocytes or astrocytoma cells expressing His-tagged MLC1 (Lanciotti et al., 2012). 2. Ouabain chromatography purified also TRPV4 (Lanciotti et al., 2012).	1. Astrocytes OE MLC1 activate calcium influx in response to hyposmosis and 4αPDD more efficiently (Lanciotti et al., 2012). 2. MLC1 also favors recycling of TRPV4 (Lanciotti et al., 2012).	1. No experiments have been performed in KO animals or astrocytes. 2. In macrophages from patients Ca^2+^ responses are also affected (Petrini et al., 2013).	It is not clear how TRPV4 activity is regulated by MLC1. It may be an indirect or a compensatory mechanism. More experimental evidence is needed.
*Kir4.1, AQP4*	1. They have been identified by co-fractionation, ouabain chromatography or pulldown, mainly in cell culture (Brignone et al., 2011). 2. AQP4 association seem to increase in hypoosmotic conditions (Lanciotti et al., 2012). 3. *In vivo* there is no colocalization by EM with these proteins in tissue (Duarri et al., 2011).	*In vitro* experiments in *Xenopus oocytes* show that MLC1 does not influence Kir4.1 channel activity (Teijido et al., 2004).	In KO animals or in brain MLC biposies there is a redistribution of AQP4 and Kir4.1, which has been interpreted as a compensatory mechanism (Dubey et al., 2015; Boor et al., 2007; Bugiani et al., 2017).	*In vitro* studies expressing these proteins are needed to prove their interaction. More evidence *in vivo* that their activity is regulated by MLC1 is necessary.
*Other proteins not related with ionic homeostasis*	1. They include: syntrophin, dystrophin, caveolin-1, ZO-1. 2. ZO-1 has been identified by coIP from brain. Co-localization has been found by EM in tissue and IF in primary astrocytes (Duarri et al., 2011). 3. GlialCAM, MLC1 and other related proteins (i.e., TRPV4) are found in caveolae (Lanciotti et al., 2010). 4. Syntrophin and Dystrophin are found by co-fractionation and pulldown (Ambrosini et al., 2008). There is no colocalization with MLC1 by EM (Duarri et al., 2011). KO of *Dystrophin*, α-*Dystrobrevin* and *Utrophin* does not affect MLC1 localization (Duarri et al., 2011).	No studies have been performed in OE mice or in transfected cells.	No changes in localization have been reported in Mlc1 or *Glialcam* KO animals (Dubey et al., 2015; Bugiani et al., 2017).	1. Some of these proteins may help anchor MLC1 and GlialCAM at specific locations, so it is logical that its localization is not affected in KO animals. 2. *In vivo*, it is not clear that MLC1 forms part of the DGC complex, since there is no colocalization between proteins of the DGC complex and MLC proteins.

*We indicate the biochemical evidence, the functional effects after overexpression or inhibition, and a conclusion of the effects*.

Classically, two approaches were followed to understand the role played by MLC and MLC-interacting proteins. One method compared the effects of overexpressing wild-type MLC1 (with or without GlialCAM) with the effects of overexpressing MLC1-containing mutations. However, an important caveat has to be stated in this method. Overexpression of Mlc1 is very toxic in mice (Sugio et al., 2017), leading to a more drastic vacuolation phenotype than in the *Mlc1* knockout mice. Also, overexpression of a protein folding mutant may cause alterations in the ER and in lysosomes leading to additional effects (Duarri et al., 2008). On the other hand, other groups explored the effects of partially or totally depleting MLC proteins. Also, some studies have functionally analyzed the activity of possible interacting proteins in animal models of the disease (knockouts or overexpression) and/or cells obtained from patients.

In general, we can conclude that many different transporters or ion channels seem to be affected in MLC. Some of the proteins affected might be directly interacting with GlialCAM/MLC1 (i.e ClC-2, Cx43, Na^+^/K^+^-ATPase), whereas others might be regulated in an indirect manner (i.e., VRAC, TRPV4). An interesting new hypothesis is that GlialCAM/MLC1 may modulate signal transduction events, and therefore, they could simultaneously influence different proteins (Lanciotti et al., 2016; Elorza-Vidal et al., 2018; Brignone et al., 2019). However, it is difficult to conclude whether the functional changes observed in these proteins are a consequence of a compensatory mechanism or a direct effect of MLC proteins. Possibly, those effects that show a different sign of change in overexpressing vs. reduced expression and that directly interact with GlialCAM/MLC proteins are probably a direct consequence of GlialCAM/MLC1 functional role. We indicate the results obtained for different proteins in Table 1.

An interesting issue is that some interactions might be constitutive, such as those between GlialCAM and MLC1 (Sirisi et al., 2014), whereas others might be regulated, such as the interaction between GlialCAM/MLC1 and ClC-2 (Sirisi et al., 2017). Hence, GlialCAM/MLC1 could be forming a protein scaffold, where different proteins may interact in a regulated manner with this protein network resulting in activity changes. As the interaction with ClC-2 was dependent on extracellular potassium, we proposed that GlialCAM/MLC1 might regulate brain homeostasis by influencing the activity and the interaction with different transporters/ion channels that could be involved in controlling brain potassium levels (Estévez et al., 2018). Apart from myelin vacuoles, defects in potassium clearance and increased excitability were described in MLC knockout mice and MLC patients (Dubey et al., 2018; Hamilton et al., 2018).

## Animal Models of MLC: Zebrafish and Mice

To understand the pathophysiology of MLC, several animal models have been generated. In mice, three different knockout lines of *Mlc1* were produced (Hoegg-Beiler et al., 2014; Dubey et al., 2015; Sugio et al., 2017), two different knockouts for *Glialcam* (Hoegg-Beiler et al., 2014; Bugiani et al., 2017), several KI mice for *Glialcam* containing dominant or recessive mutations affecting GlialCAM targeting (Hoegg-Beiler et al., 2014; Shi et al., 2019), double KO for *Mlc1* and *Glialcam* (Pérez-Rius et al., 2019), and a transgenic mouse that overexpress *Mlc1* in astrocytes (Sugio et al., 2017). It will be interesting to use *Mlc1* floxed mice to generate conditional KO specifically in astrocytes during development, or KI mice for *Mlc1* containing missense mutations as well as to obtain tissue-specific knockouts for GlialCAM. In zebrafish, an *mlc1* KO line has been generated (Sirisi et al., 2014), with a mutation that abolishes mlc1 expression *in vitro* and *in vivo*; a *glialcama* KO and a double KO for *mlc1* and *glialcama* (Pérez-Rius et al., 2019).

**Figure 2:**
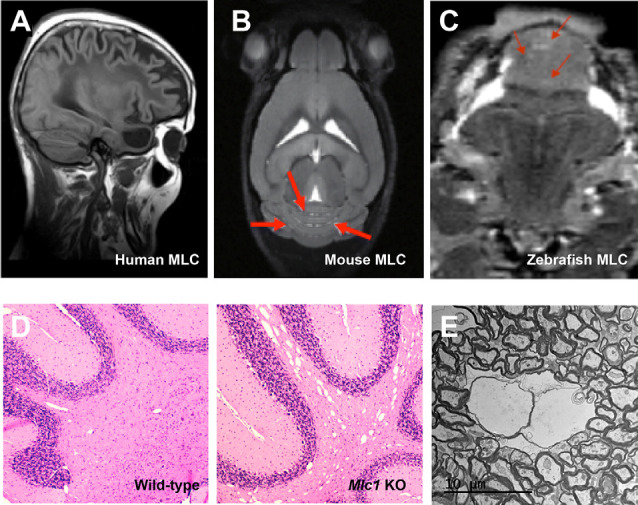
The vacuolating phenotype of GlialCAM and MLC1 in mice and zebrafish resembles the MLC phenotype observed in humans. **(A–C)** MRI images of a human MLC1 patient **(A)**, *Mlc1* knockout mice **(B)**, and *mlc1* knockout zebrafish **(C)**. Red arrows indicate areas with increased water. Reprinted by permission from Oxford University Press: Human Molecular Genetics, *Molecular mechanisms of MLC1 and GLIALCAM mutations in megalencephalic leukoencephalopathy with subcortical cysts*, López-Hernández et al. (2011b) and by permission from Oxford University Press: Human Molecular Genetics, *Megalencephalic leukoencephalopathy with subcortical cysts protein 1 regulates glial surface localization of GLIALCAM from fish to humans*, Sirisi et al. (2014). **(D)** Histology studies of cerebellar brain white matter comparing wild-type (left) with *Mlc1* KO mice indicate the presence of vacuoles in the KO mice. **(E)** EM images of vacuoles in *Mlc1* KO mice.

All knockout animals share with human MLC patients an increase in the brain water content (Figure 2), which can be easily detected by MRI or histological techniques. As in biopsies of MLC patients (van der Knaap et al., 1996), vacuoles observed in animal models using electron microscopy are intramyelinic (Figure 2). However, some differences exist between human and animal models. MRI defects are observed in humans during the first years of life, thus there is a correlation between the period where myelination is more active and the onset of vacuolization. However, it takes much more time to be seen in mice (Hoegg-Beiler et al., 2014; Dubey et al., 2015), and even longer in zebrafish (Sirisi et al., 2014). In line with these differences, expression of MLC1 and GlialCAM is also higher in humans in the first years of life and then is reduced and maintained (Dubey et al., 2015; Bugiani et al., 2017), whereas expression of both proteins increases slowly in mice, reaching its peak at adult stage (Teijido et al., 2007; Gilbert et al., 2019).

Furthermore, there are also regional differences in vacuolated areas. It is important to mention that in humans the edema is mostly found in the subcortical white matter. While mice have a much more limited subcortical white matter, vacuoles are found in the cerebellum of KO mice, although MLC1 is broadly expressed at the endfeet of astrocytes in the whole brain (Hoegg-Beiler et al., 2014). Thus, it can be speculated that MLC1 and GlialCAM physiological roles might be more important in the cerebellum than in the rest of the brain or that they might play additional roles in cerebellar Bergmann glia. Several observations support these hypotheses: (1) in mice, expression of both proteins in the cerebellum is higher than in the rest of the brain (Teijido et al., 2007; López-Hernández et al., 2011a); (2) knockout of *Mlc1* reduces total expression of Glialcam and ClC-2 in the cerebellum, whereas no changes are observed in the cerebrum (Hoegg-Beiler et al., 2014). Similarly, knockout of *Glialcam* decreases expression of ClC-2 in the cerebellum, but not in the rest of the brain. These results are in agreement with the formation of a ternary complex between GlialCAM, MLC1, and ClC-2 that stabilizes ClC-2 in the cerebellum (Sirisi et al., 2017); (3) in a brain biopsy of an MLC1 patient, lack of MLC1 causes GlialCAM mislocalization in Bergmann glia (Sirisi et al., 2014), but not in the rest of the brain (López-Hernández et al., 2011b); and (4) overexpression of Mlc1 causes a reduction in cerebellar size and Bergmann glia ectopia by an unknown mechanism in a development-dependent manner (Kikuchihara et al., 2018). Thus, we postulate that GlialCAM/MLC1 may have more importance or additional roles in the cerebellum, and this is perhaps more relevant in mice.

Different from MLC patients, the MLC mouse model containing a deletion of the first two exons of *Mlc1* (Hoegg-Beiler et al., 2014) does not display any cognitive or motor deficits and it is considered a model for the early stages of the disease. In contrast, the *Mlc1* KO where the full *Mlc1* gene was replaced by GFP showed some motor defects and higher susceptibility to develop epileptic seizures after kainate insults (Dubey et al., 2018). It remains to be determined whether these differences are due to the different targeting strategies and/or to genetic background.

One interesting result observed in animal models is the mutual dependence between GlialCAM and MLC1. Thus, in mice and zebrafish, lack of Mlc1 causes GlialCAM mislocalization but not the reduced expression (Hoegg-Beiler et al., 2014; Sirisi et al., 2014) and, in mice, lack of GlialCAM causes Mlc1 reduced expression and mislocalization (Hoegg-Beiler et al., 2014). These results are in agreement with similar clinical phenotypes among MLC2A and MLC1 patients (Hamilton et al., 2018).

Animal models have also been very useful in defining the biochemical relationships between different proteins related to GlialCAM/MLC1 cell biology. A clear example is a biochemical and functional interaction between GlialCAM/MLC1 and the chloride channel ClC-2. Lack of Mlc1 or Glialcam causes ClC-2 mislocalization (Hoegg-Beiler et al., 2014), which is completely abolished in Bergmann glia and strongly diminished at the astrocytic endfeet in the whole brain (Dubey et al., 2015; Bugiani et al., 2017). Furthermore, *in vivo* measurements of ClC-2 activity in cerebellar slices appeared linear in oligodendrocytes from wild-type mice, and inwardly rectifying from *Glialcam* or *Mlc1* knockout mice (Hoegg-Beiler et al., 2014). Other possible functional and biochemical interactions such as the VRAC channel (containing LRRC8A), Na^+^/K^+^-ATPase, Kir4.1, AQP4 or TRPV4 have not been demonstrated *in vivo*, since its localization is preserved in MLC knockout mice (Hoegg-Beiler et al., 2014; Dubey et al., 2015; Bugiani et al., 2017). Hence, these proteins might be indirectly regulated by unknown mechanisms or changes in their activity could be a consequence of a compensatory mechanism. Thus, we can conclude that many different processes might be affected by the lack of GlialCAM and MLC1. How GlialCAM and MLC1 regulate all these processes needs to be elucidated to define novel therapeutic strategies. In this respect, we explored the strategy of gene therapy as an approach to restore normal MLC1 protein levels *in vivo*, as detailed below.

## Gene Therapy for MLC

There are several major choices when developing a gene therapy strategy for a disease like MLC, involving the CNS, which can influence the efficiency of the treatment. This includes the type of vector, its route of administration, the diffusion of the therapeutic protein if can be secreted, or the need for a cell-specific promoter. Among the available gene delivery vectors, adeno-associated virus (AAV) vectors have several features that make them appropriate for brain gene transfer and one of the most promising for human trials: (i) the capacity for generating long-term gene expression, as long as 15 years in non-human primate CNS (Sehara et al., 2017) mostly as episomal form, thus, avoiding the risk for insertional mutagenesis reported with integrative vectors; (ii) the absence of toxicity associated with wild type virus (AAV vectors are classified as Biosafety Level 1), combined with the absence of wild type viral genes; and (iii) the ability to easily produce pure high-titer viruses in the laboratory.

Current technology allows us to efficiently target a localized brain region, for instance, the substantia nigra in Parkinson’s disease, in which case direct intraparenchymal injection is needed. This brings us to another important decision: the appropriate route of administration has to be approached for each disease. Direct administration of the vector to a reduced and specific area of the brain through intracranial injection is only suitable when the therapeutic protein needs to be localized in a particular structure, limiting the diffusion of the vector. Intracranial administration is extremely invasive and there is an upper limitation of dose and volume to avoid toxicity and inflammation, although it has the advantage of using lower vector volumes, thus facilitating vector production.

When the therapeutic protein can be secreted to the extracellular space, cross-correction from a reduced number of cells producing the protein can be enough to achieve therapeutic effects in most cells, independently of the transduced cell type. This is the case of some lysosomal storage diseases like mucopolysaccharidoses, where the lysosomal enzyme is partially secreted and recaptured through the mannose-6-phosphate receptor. However, in the case of MLC disease, as MLC1 and GlialCAM proteins are located in the cellular membrane of astrocytes, we might need to transduce as many astrocytes as possible.

**Figure 3:**
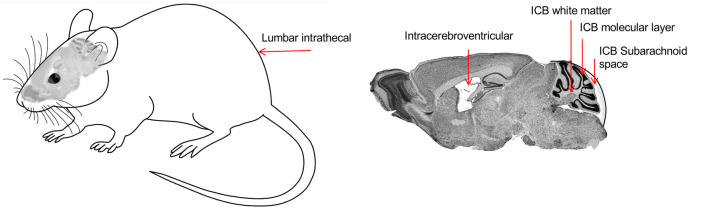
Representation of the different routes of administration to deliver viruses into the cerebellum. Lumbar intrathecal (IT), Intracerebroventricular (ICV), Intracerebellar (ICB), white matter (ICB WM), ICB molecular layer (ICB ML), and ICB subarachnoid space (ICB SB).

Indeed, many neurological disorders need a global distribution of the therapeutic protein to the whole brain or at least to large CNS structures. Moreover, some brain areas are more refractory to transduction by global routes of administration, including the cerebellum, and need a specific approach (Bosch et al., 2000). For the *Mlc1* KO mouse, where most histological abnormalities are present in the cerebellum, we explored different delivery routes involving intravenous (IV), CSF direct administration at lumbar or at the cerebellar area and directly into the cerebellar parenchyma, into the white mater or in the molecular layer (Figure 3). Ideally, IV administration of the vector would be the route of choice if a single injection could reach the whole brain, without using more interfering surgeries. However, most vectors are not able to cross the blood-brain barrier (BBB). Recently, the capacity of transducing the CNS through the circulation was shown for AAV9 and some derivates (like PHP.B; Foust et al., 2009; Deverman et al., 2016). However, PHP.B efficiency was demonstrated in C57/bl6 mouse strain (Deverman et al., 2016) but not in Balb/c mice (Hordeaux et al., 2018), and more importantly, the receptor used by this modified AAV capsid is not found in non-human primates (Hordeaux et al., 2019), which relegates the use of this vector to mouse preclinical studies. Intravenous AAV9 administration, however, can reach global areas in the cerebrum where it transduces astrocytes and neurons, as well as in the spinal cord, and is currently being applied to some clinical trials, particularly for very young children, in which the BBB is still not well closed. Encouraging results for spinal muscular atrophy and Mucopolysaccharidosis type IIIA have been reported so far (NCT02122952 and NCT04088734 in www.clinicaltrials.gov, respectively; Mendell et al., 2017). But, to achieve therapeutic levels of the transgene into the CNS when reaching it from the circulation, high titers of viruses need to be infused into the patients (>10^14^ vg/kg), with the risk of adverse secondary effects, some of which may be related to liver and dorsal root ganglia toxicity, as were seen in NHP and piglets (Hinderer et al., 2018). Moreover, the scale-up of vector production for human clinical trials is also challenging due to the high virus load needed for each patient.

**Figure 4:**
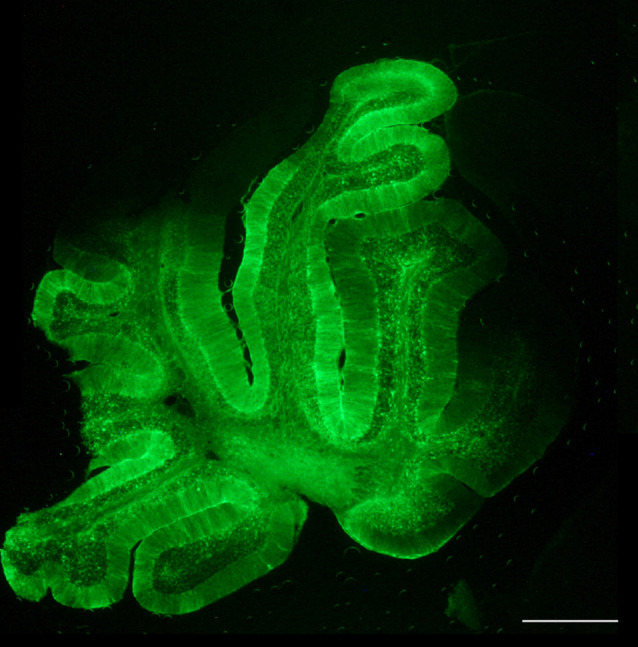
Biodistribution of AAVrh10 coding for GFP under the regulation of Glial-derived fibrillary acidic protein recombinant promoter (GFAP). Cerebellar administration of the adeno-associated virus (AAV) vector in the subarachnoid space efficiently transduces the whole cerebellum. Scale bar, 400 μm. Reprinted by permission from Springer Nature: Springer, Neurotherapeutics, *Cerebellar Astrocyte transduction as gene therapy for megalencephalic leukoencephalopathy*, Sánchez et al. (2020).

Distribution of the vector through the cerebrospinal fluid (CSF) has many advantages, as allows for a more restricted expression, compared to the IV; but better global CNS transduction, compared to intraparenchymal administration; and it requires lower amounts of viruses, thus increasing biosafety and lowering production constraints (Pagès et al., 2019). Moreover, particularly for lumbar puncture, it can be performed in an outpatient setting, as is much less invasive than intracranial administration. In the MLC mouse model, the best results were obtained by delivering the vector into the CSF by occipital subarachnoid administration, achieving a high percentage of transduction of the cerebellum without damaging the brain parenchyma, allowing for a more localized and efficient action and avoiding non-specific effects due to systemic expression (Sánchez et al., 2020). By this route, we efficiently achieved transduction in most of the cerebellar parenchyma, with the highest efficacy in the Bergmann glia, where MLC1 is highly expressed (Figure 4). Given the small volume in the cerebellar intrathecal cavity, the best diffusion was achieved by loading the virus at a very slow rate (Huda et al., 2014), which in turn may decrease the amount of virus drained to the periphery (Wang et al., 2018).

It is well known that AAV vectors inherently transduce neuronal cells, so when neurons are not the target cell and to avoid undesirable secondary effects, it is important to restrict expression to the target cell type. This is possible by combining a particular AAV serotype with the control of the transgene expression by recombinant promoters that recruit specific host cell-derived transcription factors (Lawlor et al., 2009). To design a gene therapy strategy for MLC, and taking into account that MLC1 is exclusively expressed by astrocytes in the brain, we choose an AAVrh10 vector (Sánchez et al., 2020), as it seems to transduce a larger number of glial cells in the adult brain, compared to AAV9 that showed a higher and specific tropism for neurons (Petrosyan et al., 2014). AAVrh10 was used in combination with a glial-derived fibrillary acidic protein recombinant promoter (GFAP), with high selectivity for astrocytes in the mature mouse brain (Brenner et al., 1994). Moreover, using a non-human primate serotype such as AAVrh10, we may increase the possibilities to avoid preexisting neutralizing antibodies in patients due to previous natural infections by human parvovirus, present in nature (Thwaite et al., 2015). Indeed, a high percentage of the human population is seropositive for AAV2 (Calcedo et al., 2009) and although there may be some cross-recognition between antibodies raised against different serotypes, this may decrease using viruses from other species (Thwaite et al., 2015). This is particularly important in adult patients, as they have been exposed to various infections throughout their life.

A dose-escalation study demonstrated a correlation between MLC1 expression and the degree of correction of the white matter vacuolation in the cerebellum of treated KO mice (Sánchez et al., 2020). However, wild-type animals injected with MLC1 did not show any deleterious effect, contrary to what was reported in the MLC1 over-expressing transgenic animal (Sugio et al., 2017), reinforcing the safety of the strategy and indicating that only supra-physiological levels of MLC1 may be toxic. Indeed, western blot analysis detected considerably lower levels of MLC1 in treated KO than in wild type animals, suggesting that a low amount of protein could still be therapeutic, as expected for a recessive disease.

**Figure 5:**
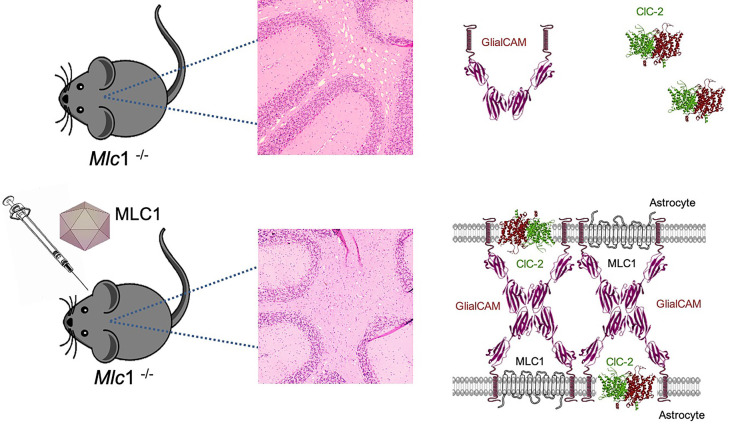
Gene therapy for MLC corrects cerebellar leukodystrophy and MLC1, GlialCAM, and ClC-2 protein complex. *Mlc1* KO mice show myelin vacuolization in the cerebellum, as a result of lack of MLC1 and mislocalization of GlialCAM and ClC-2. AAV-mediated gene therapy restores MLC1 and allows the formation of the MLC1-GlialCAM-ClC-2 protein triad, which is enough to correct the vacuolating phenotype of this model.

We tested two different approaches, first a preventive strategy, treating the animals before the beginning of the pathology and analyzing them at 8 months of age, when the cerebellar vacuolation is evident. In the *Mlc1* KO mouse, increased MLC1 expression in Bergmann glia corrected GlialCAM and ClC-2 location in this area, confirming that the ternary complex formed by the three proteins is more stable at the plasma membrane, as previously seen *in vitro* and *in vivo* (Sirisi et al., 2017). Furthermore, myelin vacuoles were significantly reduced in number and size (Figure 5). More importantly, when the animals were treated at the beginning of the symptomatology, we not only prevented the onset of the cerebellar abnormalities but also significantly reduced the vacuolating phenotype in the transduced tissues. In fact, no differences were seen between the preventive and the therapeutic group at 8 months of age.

Based on the remitting edema phenotype of MLC2B patients, who carry one dominant mutation in the *HEPACAM* gene, we hypothesized that in vacuolating leukoencephalopathies such as MLC, or as it was demonstrated for similar pathologies such as Canavan’s or Pelizaeus-Merzbacher-like disease (Georgiou et al., 2017; Gessler et al., 2017), the therapeutic correction could be achieved even in advanced stages of the disease. Importantly, in this type of disease, there is no marked neuronal degeneration, and possibly the clinical phenotype could be reversible. Indeed, the results obtained from animals treated at 15 months of age, almost 1 year after the development of the vacuolating pathology, demonstrated a similar level of correction to animals treated as asymptomatic (Sánchez et al., 2020). This is relevant for therapy, as indicates that we may have a wide therapeutic window for these patients and they might be treated at any age, while in most neurodegenerative diseases, successful treatment is conditioned to very early intervention, thus, subjected to their perinatal detection, only if they have older affected siblings, or through perinatal screening, which unfortunately is still not the case for many inherited diseases.

This study is only a proof of concept of the feasibility of gene therapy for MLC. Therefore, the translatability of this approach needs to be further confirmed as human patients display pathology through the whole brain. However, some of the most severe symptoms such as mobility problems might be partially related to cerebellar pathology, so, it needs to be tested whether expression of MLC only in the cerebellum is enough to ameliorate these symptoms. Nevertheless, other phenotypes such as autism or cognitive deterioration might be more difficult to correct since these phenotypes may be the consequence of alteration of developmental processes at very early stages. Whether the mouse model may reveal higher susceptibility to develop epileptic seizures after kainate insults (Dubey et al., 2018) and if this may be corrected after gene therapy, remains to be evaluated. Anyway, extrapolating from the tiny mouse cerebellum to the same structure in young children is challenging. So, probably we will need to deliver the virus through multiple injections, although other strategies like convection-enhanced administration should be assayed in larger animals such as non-human primates, dogs, or pigs (Lonser et al., 2015).

There are still several important concerns to be studied in this disease before thinking in a human clinical trial. It would be interesting to see if gene therapy in the *Glialcam* KO mouse model can mimic the results obtained in the *Mlc1* KO mouse. Whether GlialCAM needs to be expressed in both oligodendrocytes and astrocytes to recover normal histology, or which of these cell types has a major role in the development of the disease are questions that need to be answered. Administration of AAVs coding for GlialCAM under the regulation of an astrocyte or an oligodendrocyte-specific promoter may give us the clue for this question. Other AAV serotypes or capsid modifications to increase vector tropism for glial cells (Powell et al., 2016), using tandem promoters for both cell types (Colella et al., 2019) or detargeting the neuronal tropism of AAVs by using microRNAs may also increase the efficiency of the strategy (Colin et al., 2009).

Importantly, to follow the progression of the disease in gene therapy clinical trials we need to select appropriate readouts. Alternatively, the development of larger animal models deficient for GlialCAM or MLC1 may facilitate direct invasive procedures and to follow physiological *in vivo* approaches such as structural and functional imaging of small brain structures, which is not possible in a mouse brain, and may open the possibility to develop new *in vivo* imaging biomarkers. Moreover, larger animal models may facilitate CSF collection, and will allow finding richer behavioral phenotypes which will make more complex behavioral testing possible. Besides, it will allow assessing for a larger biodistribution of the vector in the brain and possibly identifying new parameters to follow in patients during gene therapy treatment.

## Conclusions and Perspectives

The first gene for MLC (*MLC1*) was identified 20 years ago. Although many discoveries have been made in this long journey, the exact function of the MLC1 protein is not known and there are no therapeutic interventions for MLC patients. We expect that novel results will provide wider perspectives of the pathophysiology of the disease, which will help to clarify MLC protein’s role in glial cell biology. Hopefully, this knowledge will be accompanied by advanced therapeutic solutions.

## Author Contributions

Both authors participated in the writing and revision of the manuscript. All authors contributed to the article and approved the submitted version.

## Conflict of Interest

The authors declare that the research was conducted in the absence of any commercial or financial relationships that could be construed as a potential conflict of interest.
